# Climate-Driven Synchronized Growth of Alpine Trees in the Southeast Tibetan Plateau

**DOI:** 10.1371/journal.pone.0156126

**Published:** 2016-06-03

**Authors:** Feifei Zhou, Keyan Fang, Fen Zhang, Zhipeng Dong, Dan Chen

**Affiliations:** 1 Key Laboratory of Humid Subtropical Eco-geographical Process (MOE), Fujian Normal University, Fuzhou 350007, China; 2 Key Laboratory of Cenozoic Geology and Environment, Institute of Geology and Geophysics, Chinese Academy of Sciences, Beijing 100029, China; 3 Key Laboratory of Western China’s Environmental Systems (MOE), Lanzhou University, Lanzhou 730000, China; Institute of Tibetan Plateau Research, CHINA

## Abstract

Knowledge about the spatiotemporal tree growth variability and its associations with climate provides key insights into forest dynamics under future scenarios of climate change. We synthesized 17 tree-ring width chronologies from four tree species at the high-elevation sites in the southeast Tibetan Plateau (SETP) to study the regional tree growth variability and climate-growth relationships. Despite of diverse habitats and different physiological characteristics of these species, these tree-ring chronologies shared a significant common variance in SETP. An unprecedented increase in the shared variance is found along the latter half of the 20th century, coinciding with the enhancement of the frequency of extreme rings among chronologies. It is found that minimum winter temperature tends to be the dominant climate for trees in this region. The site-specific responses in cold (1965–1980) and warm (1990–2005) intervals by means of Fuzzy Cmeans (FCM) clustering reveal that the remarkable enhancement of growth synchrony among trees mainly occur in warm conditions. This is different from previous findings indicating that increased consistence among temperature sensitive tree rings in cold periods. This may be related to the reduced temperature sensitivity of regional tree growth as winter minimum temperature is lower than a certain threshold, which is in agreement with the “principle of ecological amplitude”. In addition, it is worth noting that precipitation in June have started to restrain the tree growth since the beginning of the 1980s, which is possibly an important contributor for synchronized growth among trees in SETP.

## Introduction

Climate change exerts one of the major abiotic factors shaping the growth of the terrestrial forests via directly modulating the metabolism of the individual trees and indirectly changing the forest structures [1], which can again feedback the global climate change via modulating the local climate and the global carbon cycle [2, 3, 4]. However, current monitoring studies on the forest dynamics are often not sufficiently long to fully comprehend the response, sensitivity and adaptation of forest growth to climate change. Tree-ring records from old growth forests are ideal for such investigations due to its long duration and its close relationships with the forest biomass. In addition, tree rings are easily to collect, allowing for investigations of tree growth and climate-growth relationships across various habitats and species. Tree growth variability and its sensitivity to climate may vary across geographic areas [5, 6, 7], along altitudinal gradients [8, 9], and among tree species [10, 11].

The southeast Tibetan Plateau (SETP), together with its vicinity, is one of the most studied areas in China with a network of relatively densely distributed tree-ring chronologies developed from endemic tree species. Complex climate-growth relationships in the SETP are largely due to its large climate gradients and complex topographic features. High altitude forests in SETP are sensitive to climate variability due to harsh environment conditions that inhabit tree establishment, growth and survival. Temperature-stress appears to be one limiting factor for some coniferous forests in SETP [12, 13, 14, 15]. In addition, precipitation also tends to be one of the dominant climate limitations for tree growth in a few sites over the region [16, 17, 18]. Although tremendous efforts have been made, less is conducted about a comprehensive investigation on the spatiotemporal variations of radial growth for various tree species and their associations with changing climate in this region.

Increasing evidence has indicated that climate-growth associations are modified by a changing climate in recent decades over a variety of regions. For example, decreased sensitivity of tree growth to temperature since the 1980s, e.g. the so-called “divergence” problem, has been frequently reported from the circumpolar area in the Northern Hemisphere [19, 20, 21, 22, 23]. A change in tree-growth pattern and in the climatic response of the pine forest was found in Iberian, which was highly linked to an increase in water stress caused by warming temperature [24]. Changing relationships between tree growth and climate were observed in some sites over the northeastern Tibetan Plateau due to the recent warming climate [25]. The semi-arid forests were suffering a prolonged growth limitation accompanying accelerated spring warming in Tienshan Mountains, northwest China [26].

In this context, we hypothesize that warming and climatic variability have produced changes in tree-growth patterns, as well as in the response of alpine forests across SETP. To test this assumption, a network of pine ring-width chronologies along SETP is collected. The aims of this work are, 1) to detect temporal variability of radical growth and the possible climate determinants; 2) to investigate site-specific response to cold and warm conditions; 3) to detect growth-climate associations and their stability across time.

## Materials and Methods

### Tree-ring network

**Fig 1:**
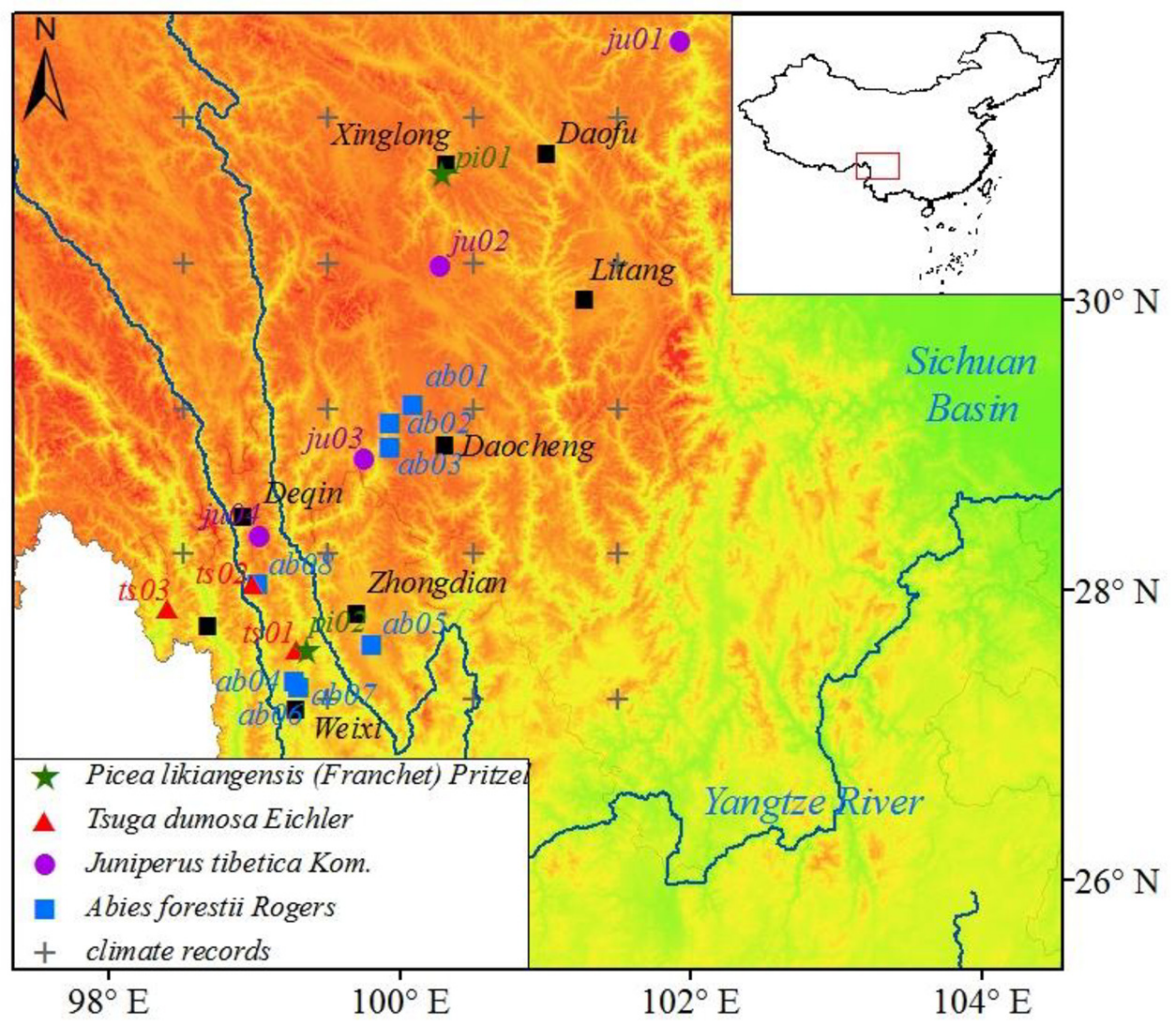
Map showing the sites of selected tree-ring chronologies, gridded climate records and major cities over SETP *Picea likiangensis (Franchet) Pritzel* chronologies are represented by green stars, *Abies forestii Rogers* chronologies by blue squares, *Juniperus tibetica Kom* chronologies by purple circles and *Tsuga dumosa Eichler* chronologies by red triangles.

Most of SETP is dominated by a highland continent climate with long, dry winter and cool, wet summer and a large diurnal range of temperature. We synthesized a tree-ring network of 17 chronologies over SETP (Fig 1; S1 Data) from the International Tree Ring Data Bank (ITRDB; http://www.ngdc.noaa.gov/paleo/ftp-treering.html). The tree-ring samples were collected from four tree species in the climate stressed sites, mainly at open forests growing on shallow soil. The dominant one is *Abies forestii Rogers* (8 chronologies), followed by *Juniperus tibetica Kom*. (4), *Tsuga dumosa Eichler* (3) and *Picea likiangensis (Franchet) Pritzel* (2). The mean segment length from chronologies of *A*. *forestii* is the longest (276.5 yr), and the average chronology length of *P*. *likiangensis* is the shortest (243.2 yr).

Biological trends were removed from the raw data by fitting straight lines or negative exponential curves to retain as much low-frequency variability as possible. Tree-ring series that could not be well fitted by the two conservative curves were detrended by a relatively stiff cubic smoothing spine curve with a 50% cutoff at around 67% of the mean segment length [27]. Tree-ring chronologies were indexed as ratios between raw measurements and the fitted growth values, which were averaged to produce a chronology based on a robust mean methodology using the program ARSTAN [28]. As the sample size generally declines in the early portion of a chronology, the subsample signal strength (SSS) was used to evaluate the most reliable time span [29]. The chronology was truncated at the year when the value of SSS became smaller than 0.85. Descriptive information on the chronologies is shown in Table 1.

**Table 1:** Descriptions of the geographic features and characteristics of the 17 tree-ring chronologies across SETP.

Site ID	Longitude (°E)	Latitude (°N)	Altitude (m)	Reliable time span (SSS>085)	No cores	Mean sensitivity	Mean segment length
***Abies forestii Rogers***							
***ab01***	100.08°	29.28°	4150	1825–2006	61	0.182	172.8
***ab02***	99.93°	29.15°	3530	1549–2006	56	0.321	282.9
***ab03***	99.93°	28.98°	3750	1697–2007	48	0.169	263.9
***ab04***	99.27°	27.37°	3050	1691–2007	27	0.216	308.2
***ab05***	99.80°	27.62°	3500	1593–2007	67	0.180	320.9
***ab06***	99.30°	27.33°	3040	1534–2007	41	0.244	366.1
***ab07***	99.30°	27.33°	3060	1791–2007	43	0.232	224.1
***ab08***	99.02°	28.04°	3200	1726–2005	19	0.242	273.4
*Juniperus tibetica Kom*							
***ju01***	101.92°	31.78°	3500	1665–2007	40	0.344	209.1
***ju02***	99.75°	28.90°	3980	1649–2007	43	0.247	278.1
***ju03***	100.27°	30.23°	4050	1595–2007	44	0.205	331.8
***ju04***	99.03°	28.37°	4260	1715–2007	42	0.159	251.2
*Tsuga dumosa Eichler*							
**ts01**	99.29°	27.59°	3150	1730–2005	49	0.249	222.1
**ts02**	98.98°	28.04°	3100	1707–2005	30	0.251	296.9
**ts03**	98.40°	27.88°	3150	1687–2005	35	0.226	286.6
*Picea likiangensis (Franchet) Pritzel*							
***pi01***	99.35°	27.58°	3240	1568–2005	36	0.249	285.2
***pi02***	100.28°	30.87°	3300	1750–2007	41	0.248	201.3

### Climate data

In total, 19 gridded climate records around the sampling sites (27°N-31°N, 99°E -102°E, Fig 1) are extracted from the CRU TS3.2 dataset, which have a spatial resolution of 1°× 1° and cover the period from 1901 to 2013 [30]. We only used the climate data since 1951 when most of the instrumental records are available for the generation of the dataset. Four climatic variables, i.e. monthly average, maximum and minimum temperature, and monthly total precipitation, are of particular interest in this study. The climate data from these grid points are significantly homogeneous through the homogeneity test [31]. Therefore, the climate data averaged from the gridded climate records were generated to represent the regional climate of the study area.

### Methods

Principal component (PC) analysis based on the correlation matrix was calculated for the overlapping period 1825–2005 to evaluate the shared variance of the chronology network. Then the first PC (PC1) with all chronologies was computed for the successive periods of 50 years lagged by 10 years in order to evaluate the temporal changes of the shared variability. For evaluation of the tree-growth variability, years with extreme wide and narrow rings (mean ± 1.5SD) were identified for each chronology.

Correlation function analysis is employed to quantify the climate-growth relationships between the regional chronology (PC1) and climate variables over a “dendroclimatic year” from the start of the previous growing season (herein May) to the end of the current growing season (September) during the period 1951–2005. Moving correlation functions (MCFs) were further used to investigate the possibly changing relationships between regional tree growth and climate data by adopting a fixed sliding window of 30 years.

To better investigate the responses of tree growth to relatively warm and cold winters (November-December-January, see Results), the corresponding tree-ring indices from each chronology under cold (1965–1980) and warm (1990–2005) conditions are extracted to create a new dataset (N = 16). Then, the fuzzy Cmeans (FCM) clustering [32, 33] was used to check for the potential partitions among these datasets. FCM is an extension of the classical K-means clustering with a concept of fuzzy logical [34]. Unlike other hard clustering methods (e.g. K-means clustering), a feature vector can belong to different groups with the degree of belongingness specified by the membership degree between 0 and 1 in the FCM theory. As we know, *it is generally impossible to come out the exclusive partitions in ecology due to the unquantifiable disturbances and the complex interactions among ecological patterns or processes* [11]. Therefore, we consider this clustering method is quite suitable for the investigations of growth response to climate change.

## Results

### Spatiotemporal tree-growth pattern

**Fig 2:**
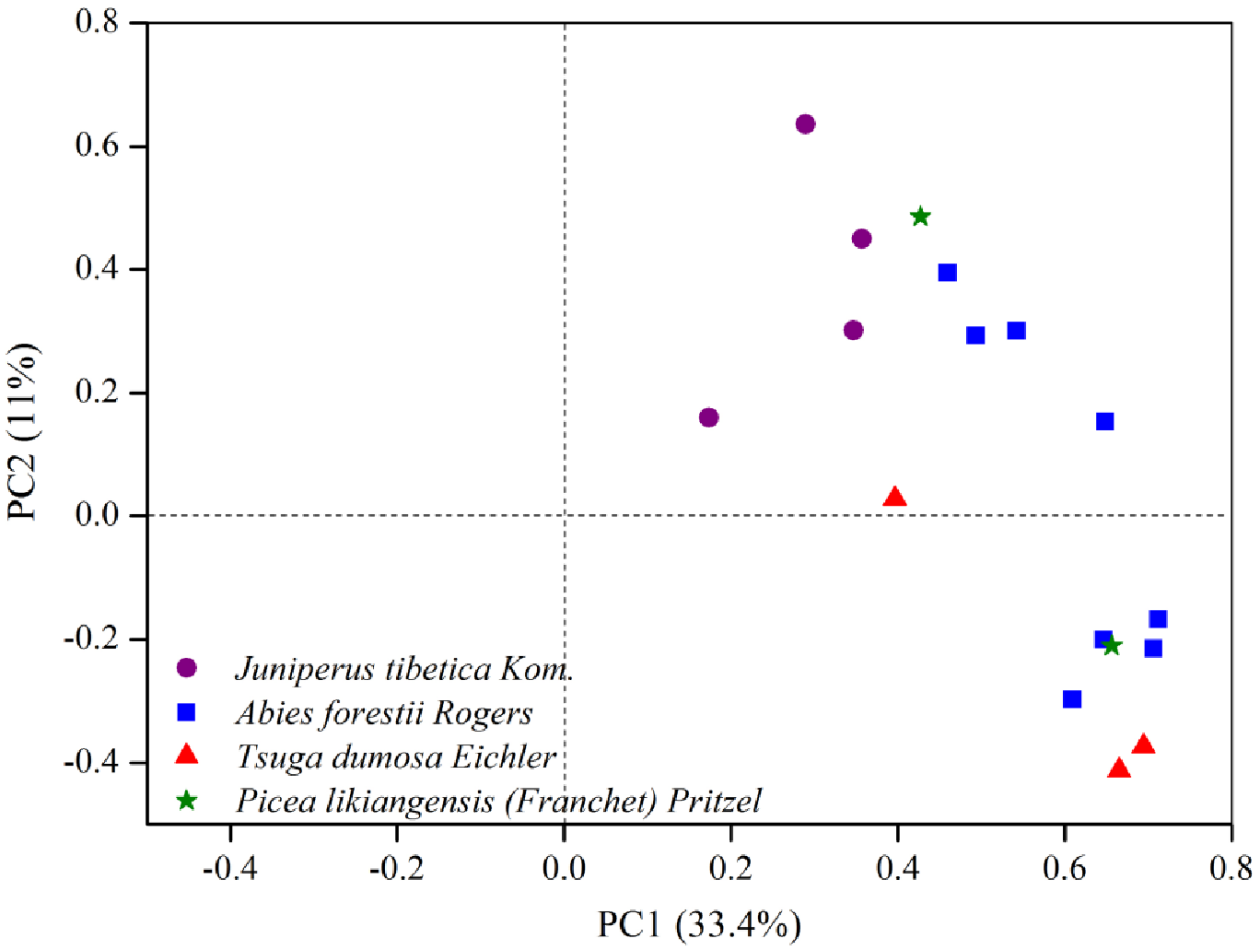
Scatter plot of the principal component (PC) loadings of the tree-ring chronology network for the common period 1825–2005 Tree species symbols as in Fig 1.

**Fig 3:**
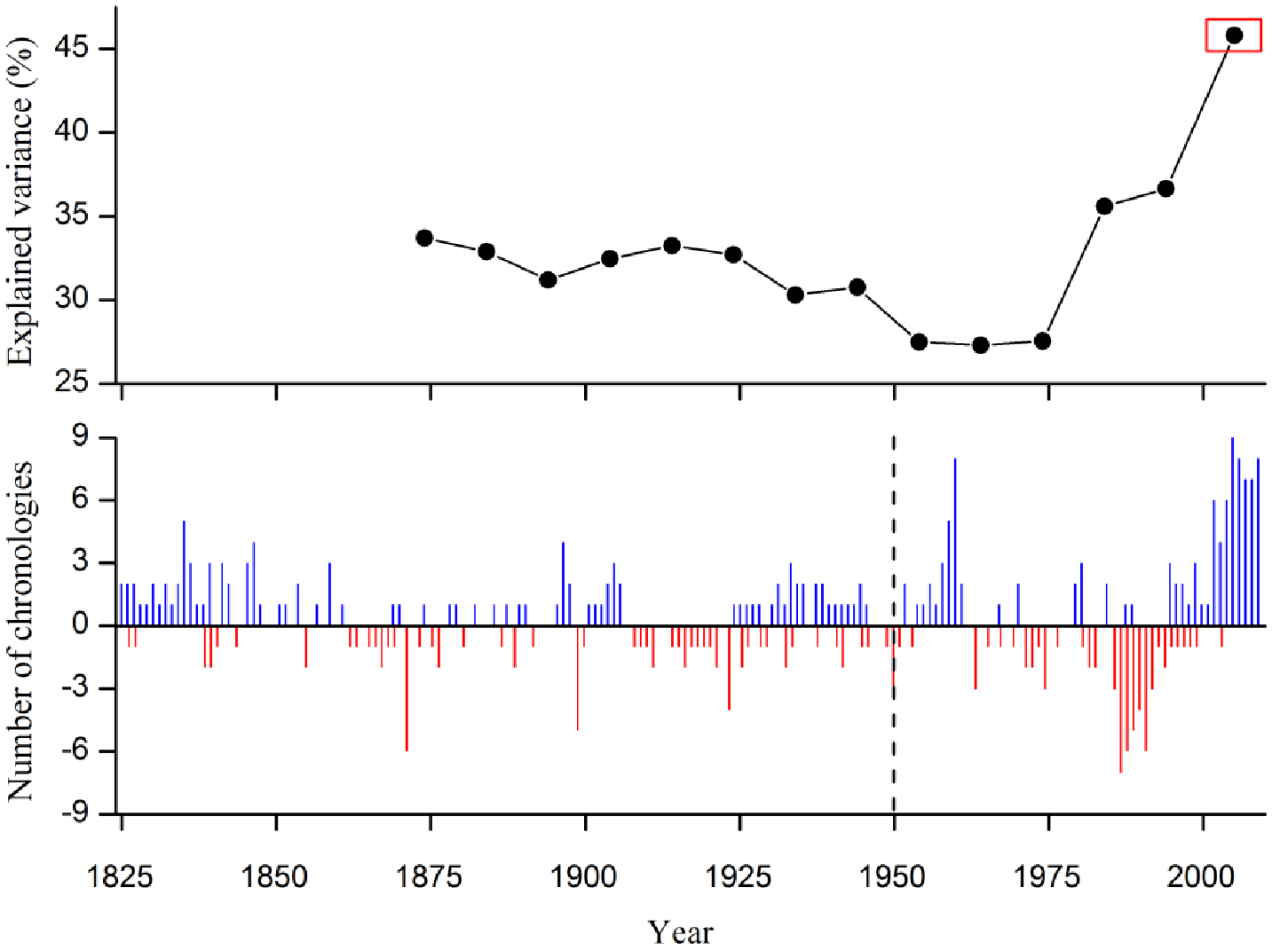
a) temporal change of the variance explained by the PC1 using a running window of 50 years lagged by 10 years; b) frequency of chronologies with extreme wide >1.5 SD, blue bar and narrow <1.5 SD, red bar during the period 1825–2005.

The PC1 and PC2 of the chronology network represent 33.4% and 11% of the total variance, respectively. As shown in Fig 2, the *T*. *dumosa* chronologies are mainly positively correlated with the PC1, while most of the *J*. *tibetica* chronologies positively correlated with the PC2. The *A*. *forestii* and *P*. *likiangensis* chronologies are relatively dispersive, part of them being the intermediate position between *J*. *tibetica* and *T*. *dumosa*. Although the chronologies show different loadings with the PC1, all of them have positive correlations with it. The evolution of the explained variance of the PC1 is shown in Fig 3a. The most distinct feature of the common variance of the chronologies is the dramatic increase along the 20th century, especially in the “1955–2005” interval when the variance explained by PC1 reached the summit at 45.8%. The number of chronologies with extremely wide (>1.5 SD) and narrow (<1.5 SD) rings has increased towards recent as shown in Fig 3b.

### Climate-growth relationships

**Fig 4:**
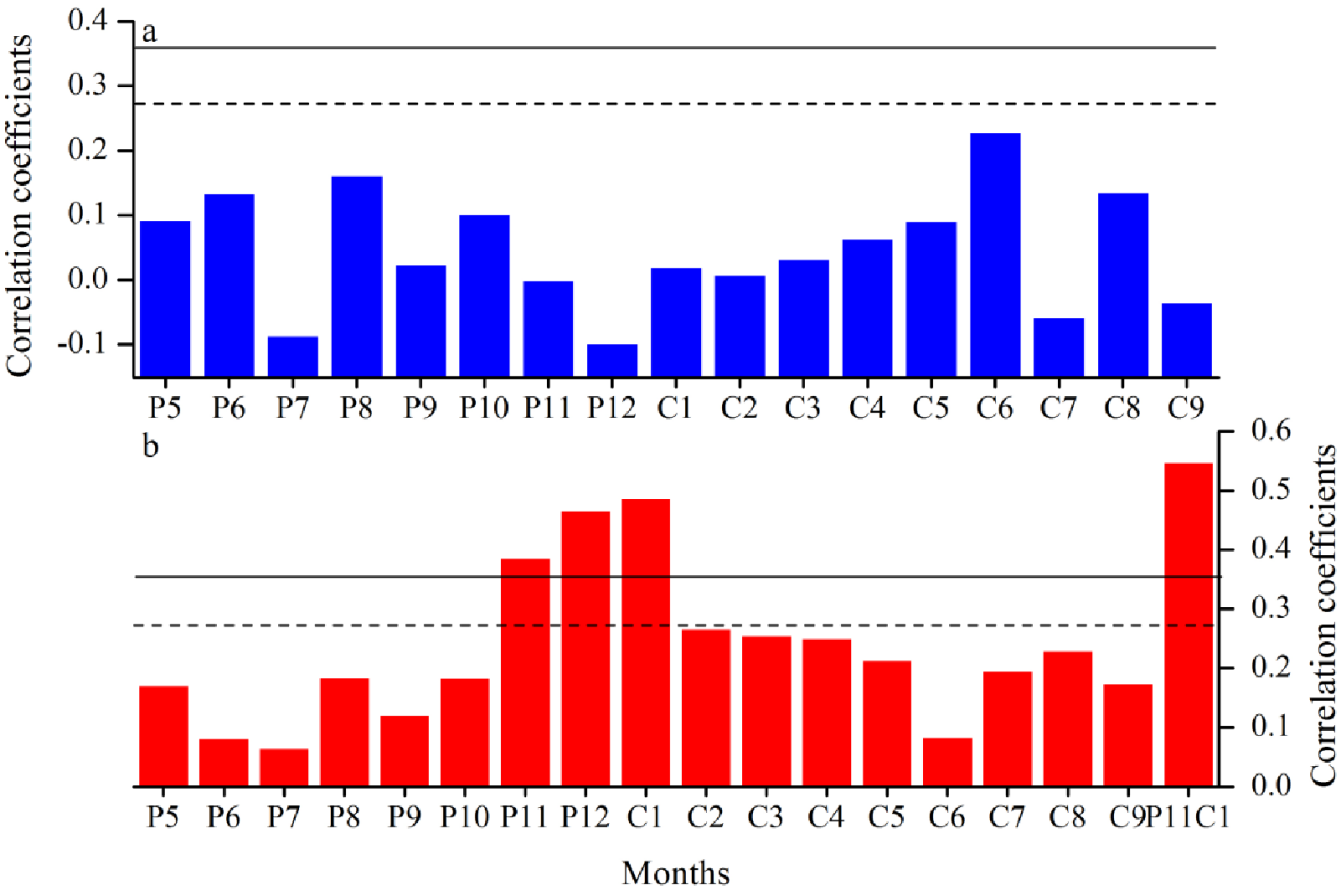
Correlations performed between the regional chronology PC1 and monthly a) mean temperature and b) total precipitation from previous May to current September during the period 1951–2005. The 95% and 99% significance levels are indicated by dash and bold lines, respectively.

No significant correlation is found between the regional tree growth (PC1) and monthly precipitation during the period from 1951 to 2005 (Fig 4a). The correlation patterns between the regional tree growth and monthly average and minimum and maximum temperature are basically similar. We herein explore the climate-growth relationships with minimum temperature that shows highest correlation coefficients (S1 Fig). As shown in Fig 4b, significant (*P<0*.*01*) positive temperature-growth associations are observed in November (0.386), December (0.464), January (0.486) during the pre-growing season. The highest correlation coefficient (0.596) is observed with November-January average minimum temperature.

### Site-specific partitions by FCM clustering

**Fig 5:**
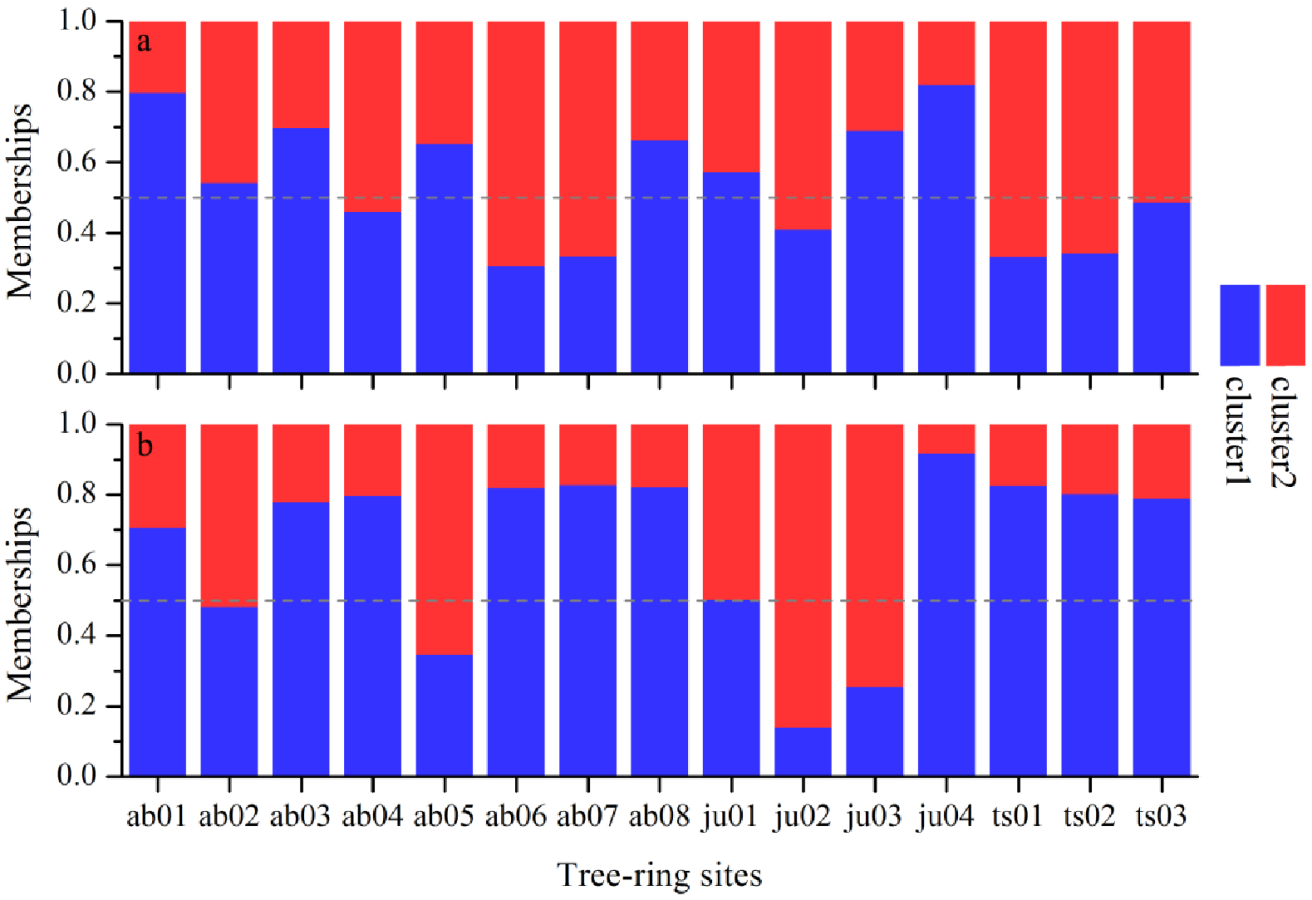
Partitions for all the indexed tree-ring widths occurring in relatively a) cold and b) warm winter years computed by the FCM clustering.

Considering the temperature-growth relationships in SETP, the results of FCM clustering are computed with the indexed tree-ring widths for all the tree-ring sites under cold and warm winter temperature conditions, respectively (Fig 5). As mentioned above, the membership of each object from the FCM clustering can be spread to any groups with an intermediate value. Under the cold intervals, the *A*. *forestii* woods at *ab01*, *ab03*, *ab05*, *ab08* sites respond in a similar way with *J*. *tibetica* woods in *ju01*, *ju03*, *ju04* sites and *P*. *likiangensis* woods in *pi01*, while trees in *ab06*, *ab07*, *ab08* sites react in a similar way with *T*. *dumosa* trees in *ju02*, *ts01*, *ts02* sites and *P*. *likiangensis* trees in *pi02* (Fig 5a). Under the warm conditions, the *A*. *forestii* woods in *ab01*, *ab03*, *ab04*, *ab06*, *ab07*, *ab08* have similar behaviors with *J*. *tibetica* woods in *ju01* and *ju04* and *T*. *dumosa* woods in *ts01*, *ts02*, *ts03* (Fig 5b). In addition, there are a few different cases of a split membership shared equally (e.g. *ts03* in cold conditions and *pi02* in warm conditions), which means that trees at these sites show no significant sensitivity to climate change.

### Growth response to climate through time

**Fig 6:**
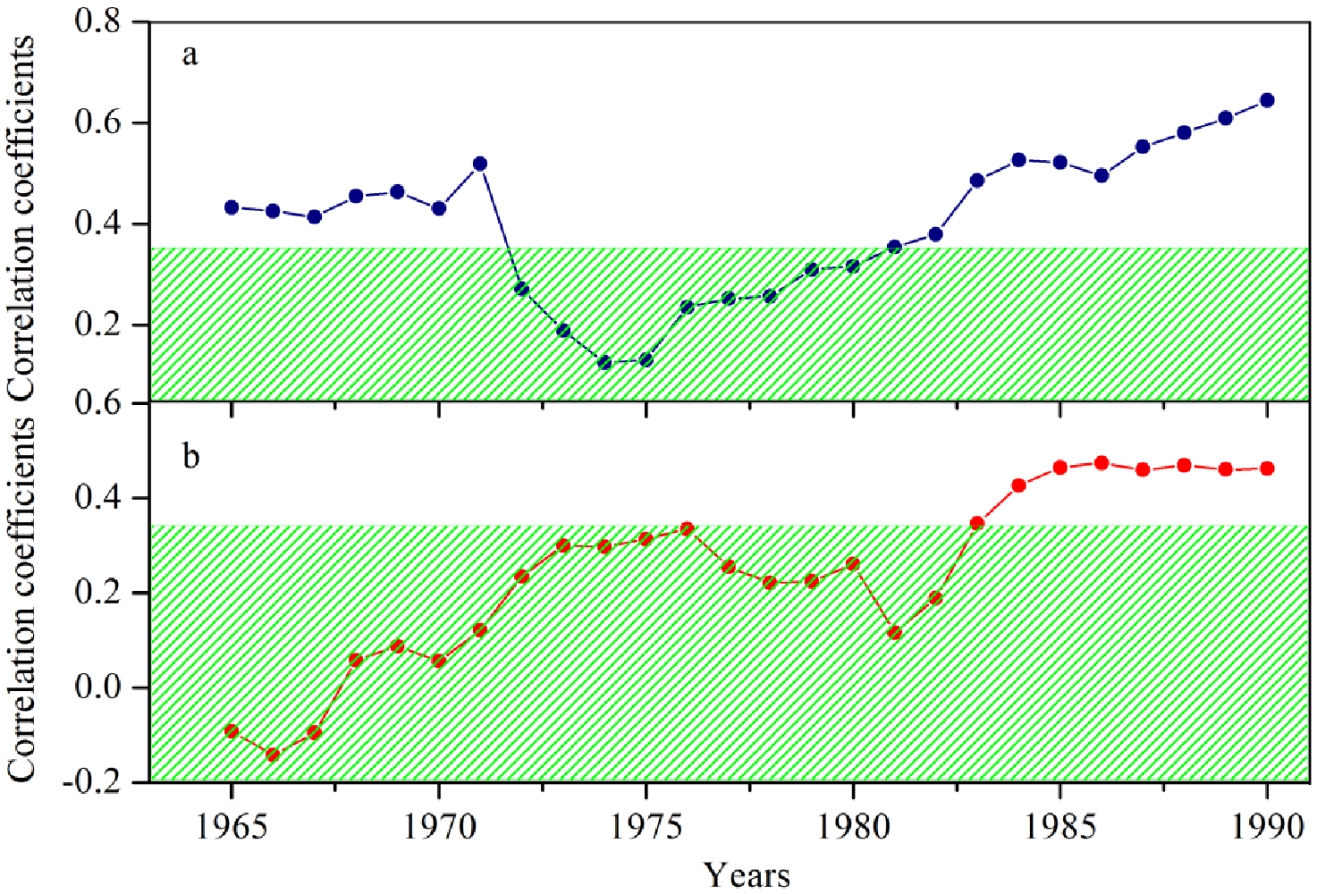
Temporal changes of the growth-climate relationships as shown by moving correlations between tree growth and a) November-January average temperature and b) June precipitation totals using a sliding window of 30 years assigned to the center year of the window. The shaded areas are the 95% confidence limits.

The MCFs reveal that the climate-growth interactions in SETP have varied through time (Fig 6). It is notable that correlation values between radial growth and November-January minimum temperature were positive, but lost its significance during the “1982–1990” period (Fig 6a). In addition, regional tree growth in this region has been significantly positively correlated to precipitation in June since approximately the time interval of “1983–1990” (Fig 6b). These marked temporal shifts in climate-growth interactions appear not be related to the effect of stochastic processes, as revealed by the Gershunov test [35].

## Discussion

### Temporal variability of radial growth

The ring-width chronologies in this study were derived from a wide range of forests of four different species in high-elevation sites across SETP. Despite of this, the PC1 held a significant percentage of common variance for these tree-ring chronologies (Fig 2), suggesting that these tree rings contain a large-scale climatic signal that diluted in individual sites. The variance held in common by these chronologies was not stable through time and increased remarkably along the second half of 20th century (Fig 3a). Higher similarities among chronologies generally increase under more limiting climatic conditions [24]. In addition, the frequency of extreme rings also rose during the recent decades (Fig 3b), suggesting more years in which climatic conditions impacted tree growth. It seems that climate may be the main cause for growth pattern changes across species in the study area and the casual link between them can be observed via the establishment of climate-growth relationships.

### Limiting role of winter temperature on tree-growth

Temperature-stress in the pre-growing season appears to be the prevailing climate limitation for tree growth at these sites in SETP as inferred from positive correlations with winter minimum temperature (Fig 4). Warm winters can prevent chilling damage to tree roots and promote organic grains for the onset of cambial activities when tree leaves are not frozen [14]. Conversely, cold conditions may also lead to the thickening of forest frozen soils and thus delay the start of the growing season [14, 36], causing the formation of narrow rings. Similar climate-growth pattern during the pre-growing season was widely documented in many regions, such as northeastern Tibetan Plateau [36], Changbai Mountains in the northeast China [14], Dabie Mountains in the lower reaches of Yangtze River [37] and several alpine areas in South China [38].

### Change in climatic response

The increase in consistent growth pattern among trees often occurs when the stress from climate is enhanced [27, 39, 40, 41]. For instance, more consistent tree growth is generally observed for drought-sensitive tree rings in drought years [9, 10, 42, 43] and for the temperature-sensitive trees in cold years [5, 13, 44, 45]. However, this study gives us a different picture, which reveals that more temperature-sensitive trees over SETP show higher synchronized growth in warm conditions (Table 2 and Fig 5). This finding highlights that the increase in the sensitivity of tree growth to climate may be driven by recent rapid warming, which could cause the frequent extremes among these chronologies.

**Table 2:** Result statistics of the FCM clustering. Group 1 and Group 2 stand for the number of the number of objects within each cluster; p-F symbols pseudo F statistic, the higher the value the better the partition. N is the number of years with significant differences between the clusters computed after the partitions.

Temperature	Cluster Distance	Group 1	Group 2	p-F	N
**Cold intervals**	0.0719	9.058	7.942	11.582	12
**Warm intervals**	0.2489	10.774	6.226	14.659	15

**Fig 7:**
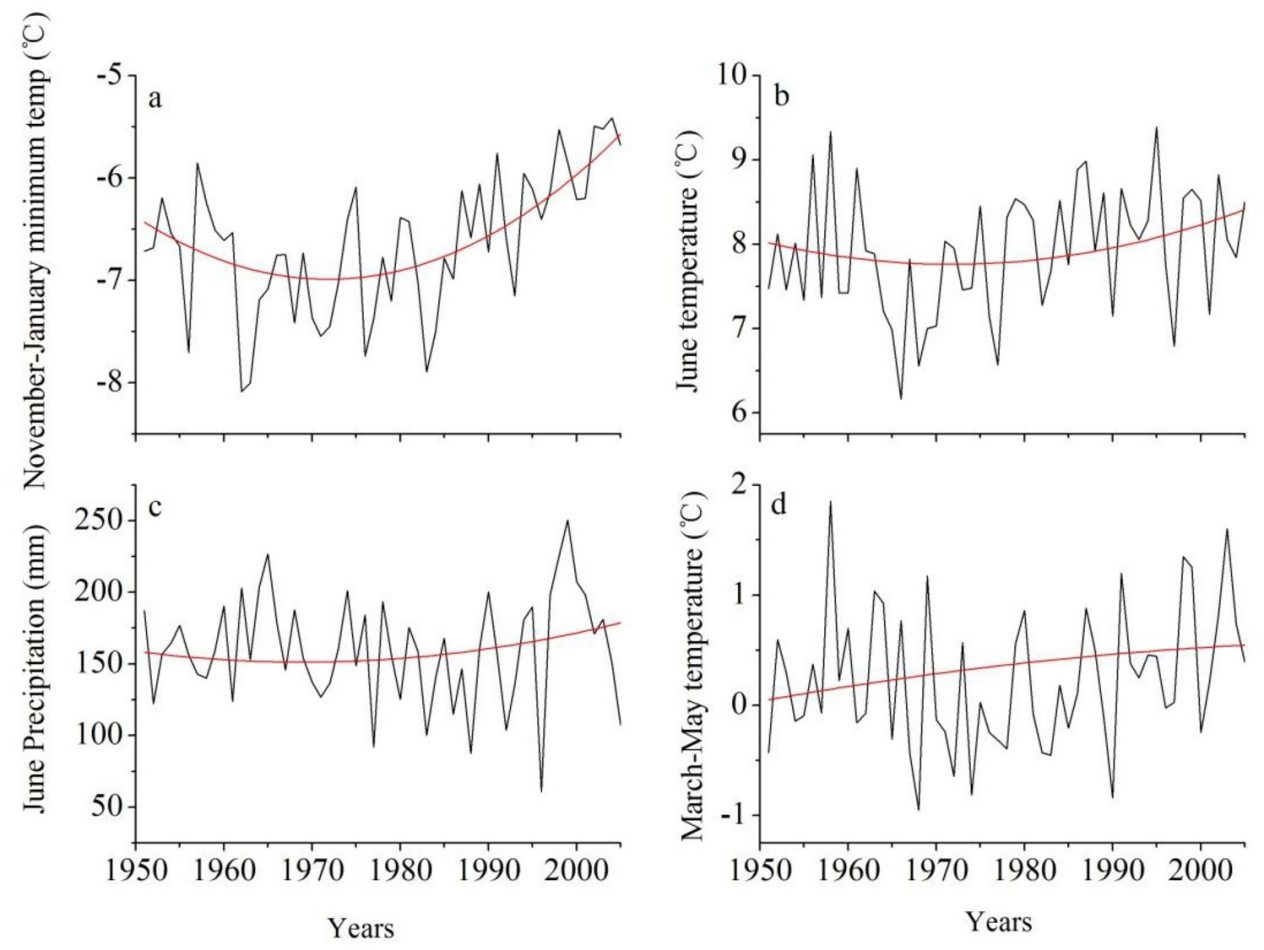
The instrumental record (black line) and EEMD-based nonlinear trend (red line) of a) November-January temperature, b) June temperature, c) June precipitation and d) March-May temperature over SETP during the period from 1951–2005.

**Fig 8:**
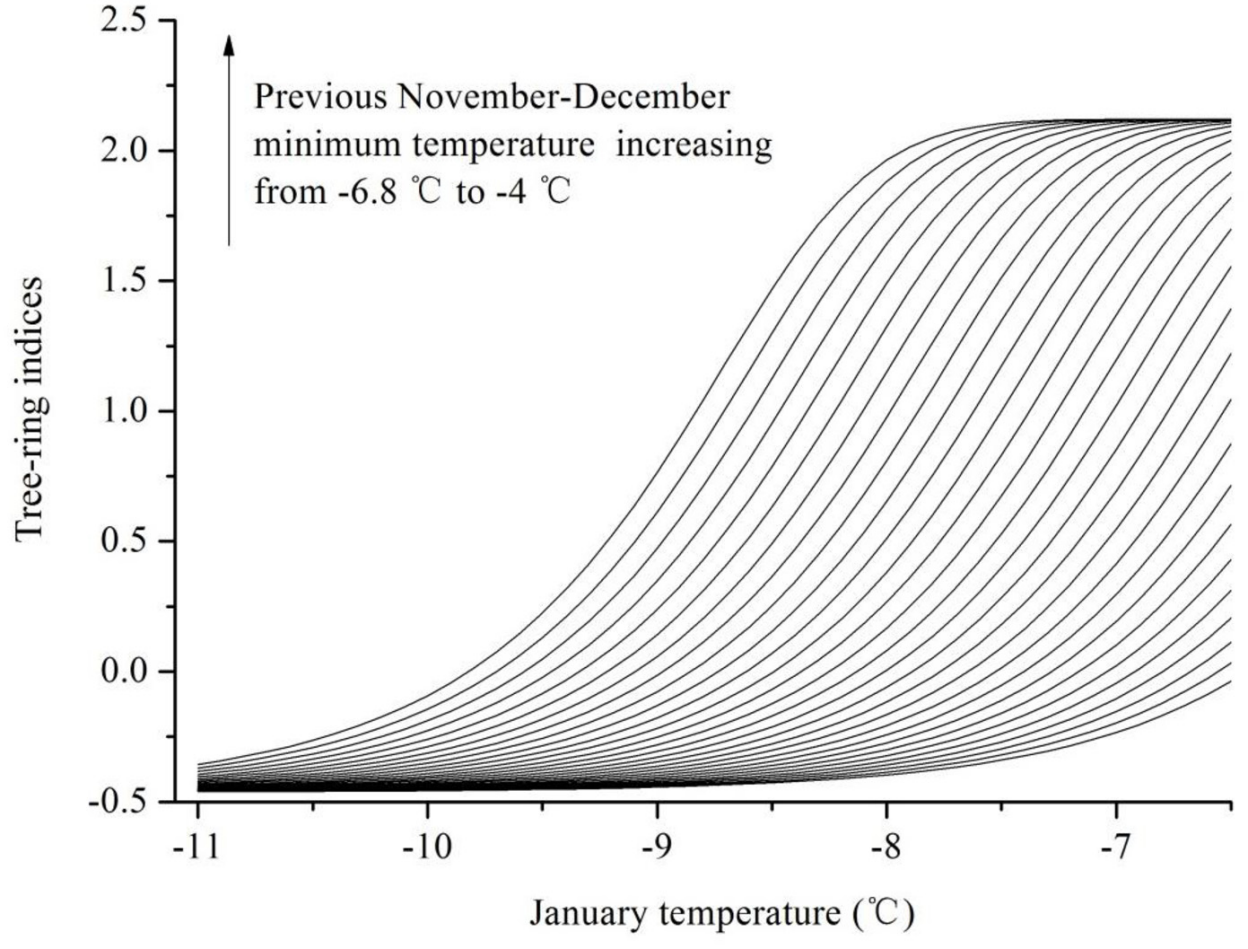
Artificial neural network (ANN) simulates tree-ring indices from January average temperature increasing from -11°C to -65°C associated with previous November-December average temperature increasing from -68°C to -4°C during the period 1951–2005.

Low sensitivity to winter temperature for tree growth was observed during the cold 1960s-1970s period (Fig 6a), indicating a potential nonlinearity in climate-growth relationships. Although temperature has been experiencing significant warming at an unprecedented pace in the past century over the most parts of the Northern Hemisphere [46], winter temperature in SETP maintained a low level at this period based on the analysis of ensemble empirical mode decomposition (EEMD) [47] (Fig 7a). Tree-ring-based temperature reconstruction from nearby have also revealed that the 1960s-70s period was one of the coldest intervals since the end of the Little Ice Age in SETP [48, 49]. In order to test the nonlinearity between trees and climate, we employ the feed-forward backward-propagation artificial neural network (ANN) method [50, 51, 52, 53] to examine the climate-growth relationships. Detailed introductions to the ANN method and the design of the network are given in S1 Text. We herein trained the ANN model by simulating regional tree growth under different combinations of previous November-December average temperature and current January temperature. When winter minimum temperature is roughly lower than -7°C, the strength of the linear temperature-growth relationship decreases remarkably (Fig 8). The decreased linear temperature-growth relationships in cold periods explain the reduced temperature sensitivity and thus less consistency among tree growth in cold periods. The ANN modeling result is in agreement with the “principle of the ecological amplitude” [53], i.e. temperature-stressed trees show limited ability in recording extreme cold conditions.

In addition, it is not negligible that regional tree growth has elevated its sensitivity to precipitation in June since approximately the early 1980s (Fig 6b), indicating that moisture conditions during the growing season started to play a limiting role on tree growth in SETP over the recent decades. Previous studies have suggested that the radial increment of trees nearby the study region generally reaches the peak in May and June [7, 54], thus a large amount of soil water supply is of a necessity to maintain the high level of cell division and enlargement. However, the warmer conditions in June since 1970s without concurrent significant increase in precipitation (Fig 7b and 7c) could have made a large effect on water balance in SETP via enhancing the evapotranspiration. In addition, regional tree growth could have benefited from the seasonal frozen soil when precipitation during the growing season is not available. Earlier snowmelt and increased evaporation coupled with the warmer spring (Fig 7d), together with the poor water-holding capacity of the thin soil, have possibly reduced the regional soil water content remarkably. Therefore, precipitation availability in June tends to be increasingly more crucial to tree growth. Similar warming-induced shifts of climate-growth associations have been prevalent in the Northern Hemisphere circumpolar area and many mountainous regions in the mid-latitudes [4, 22, 23, 25, 55].

## Conclusions

We have developed 17 tree-ring width chronologies across SETP from four endemic tree species. Despite of the diversity of species and habitats, these chronologies shared a significant common variance (PC1) that can express the large-scale climatic signal in the study area. An unprecedented increase in the shared variance is detected during the latter half of the 20th century. Regional tree growth is mainly limited by winter temperature during the pre-growing season. However, consistence among tree-growth and the temperature-growth relationships become strong (weak) in warm (cold) periods based on the FCM clustering analysis. This is different from previous findings that indicated increased (decreased) consistence among temperature sensitive tree rings in cold (warm) periods. This suggests the presence of nonlinear climate-growth relationships. It is found that tree growths are less sensitive to minimum temperature in freezing winters. Additionally, June precipitation shows significant correlations with tree growth since the beginning of the 1980s, which is possibly related to elevated evaporative demands and degradation of the seasonal frozen soil during the growing season due to the recent warming. Both recent warming in winters and spring drought may induce the coherent growth pattern among trees over SETP.

## Supporting Information

S1 Data Tree-ring data. (ZIP)

S1 Fig Climate-growth relationships. Correlations between the regional chronology PC1 and monthly a) mean temperature (green), b) maximum temperature (red) and c) minimum temperature (blue) from previous May to current September during the period 1951–2005. The 95% significance level is indicated by dash lines.
(TIF)

S1 Text The design of artificial neural network. (DOC)
