# Supplementary figures and images for: Climate-Driven Synchronized Growth of Alpine Trees in the Southeast Tibetan Plateau
Source: PLoS One. 2016 Jun 3;11(6):e0156126. doi: 10.1371/journal.pone.0156126 (PMC4892591; doi:10.1371/journal.pone.0156126)

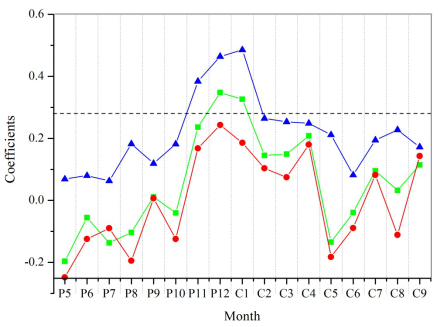

Supplement: S1 Fig — Correlations between the regional chronology PC1 and monthly a) mean temperature (green), b) maximum temperature (red) and c) minimum temperature (blue) from previous May to current September during the period 1951–2005. The 95% significance level is indicated by dash lines. (TIF) [file pone.0156126.s002.tif]
