# Supplementary material for: Climate-Driven Synchronized Growth of Alpine Trees in the Southeast Tibetan Plateau
Source: PLoS One. 2016 Jun 3;11(6):e0156126. doi: 10.1371/journal.pone.0156126 (PMC4892591; doi:10.1371/journal.pone.0156126)
Supplement: S1 Text — (DOC) [file pone.0156126.s003.doc]

**Designs of the Artificial Neural Network (ANN)**

The ANN model includes (1) the input layer with November-December and January temperature, (2) the hidden layer with two neurons, and (3) the output layer of the PC1 indices. The nonlinearity was employed by using the nonlinear transfer functions (logistic sigmoid function) between the input and hidden layers and the complex dimension of ANN. The transfer function between the hidden and output layers is a linear function. 60% of randomly divided climate data (calibration dataset) were fed in the model to adjust the weights between three layers in order to minimize the sum of squared errors between estimated and actual data during the training process, using the Levenberg-Marquardt algorithm. The ANN model was validated using 20% of the randomly-selected data and was tested by 20% of the independent data. The trained ANN was used to simulate tree growth for some scenarios of input monthly climate variables and to examine the nonlinear climate-growth relationships (Zhang et al., 2000; Fang et al., 2013).

The ANN modeling is implemented using the MATLAB toolbox. The network includes three layers with one hidden layer with two neurons. The training function in the toolbox is TRAINLM, the learning function is LEARNGD, and the model performances were examined using the MSE (mean squared error) statistics. The data were randomly selected for training data (60%), validation (20%) and testing (20%). The training parameters used in the MATLAB toolbox are as below.

Training parameters

Show: 25

Show window: true

Show command line: false

Epochs: 1000

Time: inf

Goal 0

Max-fail 6

Mem-reduc 1

Min-grad 1e-010

Mu 0.01

Min_grad:1e-010

Mu:0.001

Mu_dec: 0.1

Mu_inc: 10

Mu_max: 10000000000

**References**

Fang K, Gou X, Chen F, Frank D, Liu C, Li J, Kazmer M (2012) Precipitation variability during the past 400 years in the Xiaolong Mountain (central China) inferred from tree rings. Climate Dynamics 39: 1697-1707.

Zhang Q, Hebda J, Alfaro I (2000) Modeling tree-ring growth responses to climatic variables using artificial neural networks. Forest Sciences 46:229–239.
